# Amygdala Engagement in Response to Subthreshold Presentations of Anxious Face Stimuli in Adults with Autism Spectrum Disorders: Preliminary Insights

**DOI:** 10.1371/journal.pone.0010804

**Published:** 2010-05-25

**Authors:** Geoffrey B. C. Hall, Krissy A. R. Doyle, Jeremy Goldberg, Dianne West, Peter Szatmari

**Affiliations:** 1 Department of Psychiatry and Behavioural Neurosciences, Faculty of Health Sciences, McMaster University, Hamilton, Ontario, Canada; 2 The Brain-Body Institute, St. Joseph's Healthcare, Hamilton, Ontario, Canada; 3 Offord Centre for Child Studies, McMaster University, Hamilton, Ontario, Canada; University of Groningen, Netherlands

## Abstract

Current theoretical models of autism spectrum disorders (ASD) have proposed that impairments in the processing of social/emotional information may be linked to amygdala dysfunction. However, the extent to which amygdala functions are compromised in ASD has become a topic of debate in recent years. In a jittered functional magnetic resonance imaging study, sub-threshold presentations of anxious faces permitted an examination of amygdala recruitment in 12 high functioning adult males with ASD and 12 matched controls. We found heightened neural activation of the amygdala in both high functioning adults with ASD and matched controls. Neither the intensity nor the time-course of amygdala activation differed between the groups. However, the adults with ASD showed significantly lower levels of fusiform activation during the trials compared to controls. Our findings suggest that in ASD, the transmission of socially salient information along sub-cortical pathways is intact: and yet the signaling of this information to structures downstream may be impoverished, and the pathways that facilitate subsequent processing deficient.

## Introduction

The autism spectrum disorders (ASD) are a complex group of disorders defined by deficits in social interaction, communication and a pattern of circumscribed interests and repetitive behaviours [1] Fundamentally neurobiological in origin, ASD is marked by early developmental onset [2] and is among the most heritable of psychiatric disorders [3], [4].

Often considered core to ASD are the impairments in social interaction as other symptoms are observed more heterogeneously and share traits with a range of neuropsychiatric disorders such as primary language delay disorders and mental retardation syndromes [5]. As a consequence, formulations regarding the underlying neuropathological changes central to the disorder have emphasized loss of amygdala function [5], [6].

The amygdala has long been accepted as the fast-acting, social appraisal centre of the limbic system. It plays a critical role in emotional arousal to fearful [7], [8], threatening and uncertain external events [9], [10]. There is converging evidence that the amygdala also plays a central role in the perception, interpretation and recognition of emotion in faces [7], [11]–[15] and may function to signal the social salience of emotional displays [16], [17]. Several current theoretical models of autism link social and emotional impairments of the syndrome to amygdala dysfunction [6], [18], [19]. However, the extent to which amygdala functions are compromised in ASD has become a topic of debate in recent years [20]. A number of postmortem studies have shown increased cell packing density in the amygdalae of individuals with autism [21], [22]. However, a recent study failed to replicate differences in cell packing, but instead identified fewer neurons in the amygdalae of individuals with autism [23]. Similarly, some volumetric studies have shown increased amygdala volumes in autism [24], [25], while others have not reported the same [26]. Functional magnetic resonance imaging (fMRI) studies have also produced discrepant findings, with some studies identifying amygdala hyporesponsiveness during the discrimination of emotional states [27], [28], and others reporting normal amygdala activation during cognitive tasks involving facial familiarity judgment and perceptual and linguistic emotion labeling [29], [30].

These discrepancies may result from a number of different factors such as the inherent heterogeneity of ASD, methodological differences between studies, comorbid diagnoses and atypical responses to environmental input [31]. Consequently, a systematic approach is called for to thoroughly investigate the social deficits in autism and test specific hypotheses regarding their underlying neurodevelopmental substrates.

An exemplary study by Dalton et al.,[32] in investigated the relationship between gaze fixation and brain activation in individuals with autism during the viewing of human faces. They found that eye fixation time was positively correlated with amygdala and fusiform activation in individuals with autism. These findings suggest that the hyporesponsiveness of the amygdala observed in other studies may not have resulted from a failure to assign emotional relevance to the stimuli and the consequent reduced salience of such stimuli for the individual with autism [27]. Amygdala hypoactivation may reflect a compensatory response to the brief over-arousal produced by such social stimuli [32]. This suggests that temporally, the amygdala in ASD may be initially as responsive or even hyper-responsive to social cues as compared to that of typically developed individuals. Indeed, the automatic, stimulus driven, recruitment of the amygdala may be intact in ASD.

Dalton's work also highlights the importance of the interplay between the amygdala and the broader affective network that subserves social cognition, including the fusiform, orbitofrontal and superior temporal regions of the brain. Here, the amygdala provides a rapid response bias as to the potential threat of a social situation, the fusiform assembles a perceptual representation of faces thus providing for recognition, and the temporal regions extract socially relevant information like eye gaze and facial movements. To date, while disruptions in each of these regions have been identified in functional imaging studies of autism, the integration of these regions has been largely overlooked. It is important, therefore, to begin to explore the relative contribution each region makes to the social cognition deficit in autism.

Imaging research in typically developed adults has shown that the amygdala is engaged in the implicit or automatic processing of emotional expressions [28], [33]. The amygdala can be engaged subconsciously by presenting images of facial emotion very rapidly such that they fall outside conscious awareness [34], [35]. LeDoux [36] has suggested that fear-related responses are sub-served by a direct subcortical pathway linking the thalamus to the amygdala, thus permitting threat stimuli to be processed rapidly, automatically, and outside conscious awareness. A secondary route engages unimodal and multimodal association cortices as well as subcortical hippocampal-amygdalar networks and is thought to be responsible for slower conscious appraisal of stimuli and the initiation of behavioural responses.

The rapid subcortical route has adaptive survival value because it permits a reflexive response to occur prior to a more thorough conscious appraisal of the threat stimulus. Behavioural and autonomic responses indicative of processing along the subcortical route have been recorded using backward masking paradigms. These rapidly present anxious face stimuli outside of conscious awareness [37], [38]. This paradigm also provides a unique opportunity to examine the proficiency of the subcortical route into the amygdala. In our design of this pilot we wished to examine the engagement of the amygdala across two participant groups, ASD and controls. Consequently, we report here the results of a functional imaging study that used backward masking and the subthreshold presentation of anxious face stimuli to examine amygdala activation in high functioning adults with ASD and controls. Importantly, it is well established in the literature that the most robust engagement of the amygdala is observed with the presentation of anxious faces [33], [7]. Therefore we opted to present many face trials using only anxious face stimuli in the backward masked condition so that we would better differentiate the activation for our ASD participants from that of controls. As this represents a departure from a more conventional design that would apply rapidly presented neutral faces as a baseline we conducted a small test series of scans to demonstrate that our design and anxious face stimuli were indeed associated with heightened engagement of the amygdala.

## Methods

### Participants

The study was approved by the McMaster University Health Sciences Research Ethics Board and conducted in accordance with its guidelines. Written informed consent was obtained from all participants. Participants were excluded from study if they had a previous or current neurological disorder, head trauma, substance use, or medical contraindications to magnetic resonance imaging. Participants were 12 high functioning male adults with ASD (x = 31.8 years old; range 19–52 years) and 12 typically developed male controls (x = 32 years old; range 19–57 years). The two groups did not differ significantly in age (t(22) = −0.04, p<0.97). The study groups were matched on age, sex and non-verbal IQ (Stanford Binet) (see Table 1.). No significant differences in were identified between the two groups in Non-verbal IQ (t(18) = 2.10, p<0.16). All participants with ASD carried a clinical diagnosis of an ASD (i.e., Autism, Asperger syndrome or PDDNOS). The Autism Diagnostic Observation Schedule - Generic (ADOS-G) was carried out on 11 of the 12 participants confirming the classification of an ASD. The remaining participant was unavailable for ADOS-G testing. Participants received a small monetary remuneration for their participation.

**Table 1:** Demographic Data.

	Autism Spectrum Disorder	Healthy Controls
Number of Participants	12	12
Age (mean, range years)	31.8 (19–52)	32.0 (19–57)
NonVerbal IQ * (n, mean ± S.D.)	96.0±20.5	106.6, ±11.54
Social Responsiveness Scale (Constantino et al., 2004)	114.2±19.9	
Mind in the Eyes Test (Baron-Cohen et al., 2001)	21.5±2.8	
ADOS (Communication) (mean ± S.D.)	5.22±1.39	
ADOS (Social Reciprocity) (mean ± S.D.)	10.33±2.73	

ADOS, Autism Diagnostic Observation Schedule [39]: * missing IQ data on 2 Ss in each group.

Prior to the scan day, adults with ASD were given an orientation that included an outline of the study, exposure to the routines associated with having an MRI (MR safety screening, changing out of street clothes, ear protection, etc.) and a quick 3 minute structural scan. After the scan they were debriefed individually, and none expressed concern or reluctance regarding continued participation in the study. The separate anatomic scan session and orientation were carried out to minimize the likelihood that anxiety associated with the scan procedures would contribute to the fMRI findings. In separate individual sessions, the performance subtests of the Stanford Binet were administered to the adults with ASD. In a single session prior to their MR scans each control subject was given a similar study orientation, tour of the MR facility, and Stanford Binet testing. In every case subject orientation was scheduled as close to the scan date as possible.

### Experimental Task

In order to present a continuous series of non-repeating face images, a large set of face stimuli were generated from two standardized emotion face batteries [40], [41]. Each face image was adjusted for size, contrast and luminosity. Next a single face image was selected as a standard, and then all faces in the battery were shifted so that the location of the pupils of the eyes in each face aligned with the pupils in the standard. Hair and background details were then occluded by an 18% grey oval cut-out. The same cut-out was applied to each face. This preparation of images was necessary to ensure that the transition from one image to the next would not be signaled by changes in the location of each face. The final face battery consisted of 64 faces netural in emotion (32 male, 32 female) and 64 anxious faces (32 male, 32 female).

**Figure 1:**
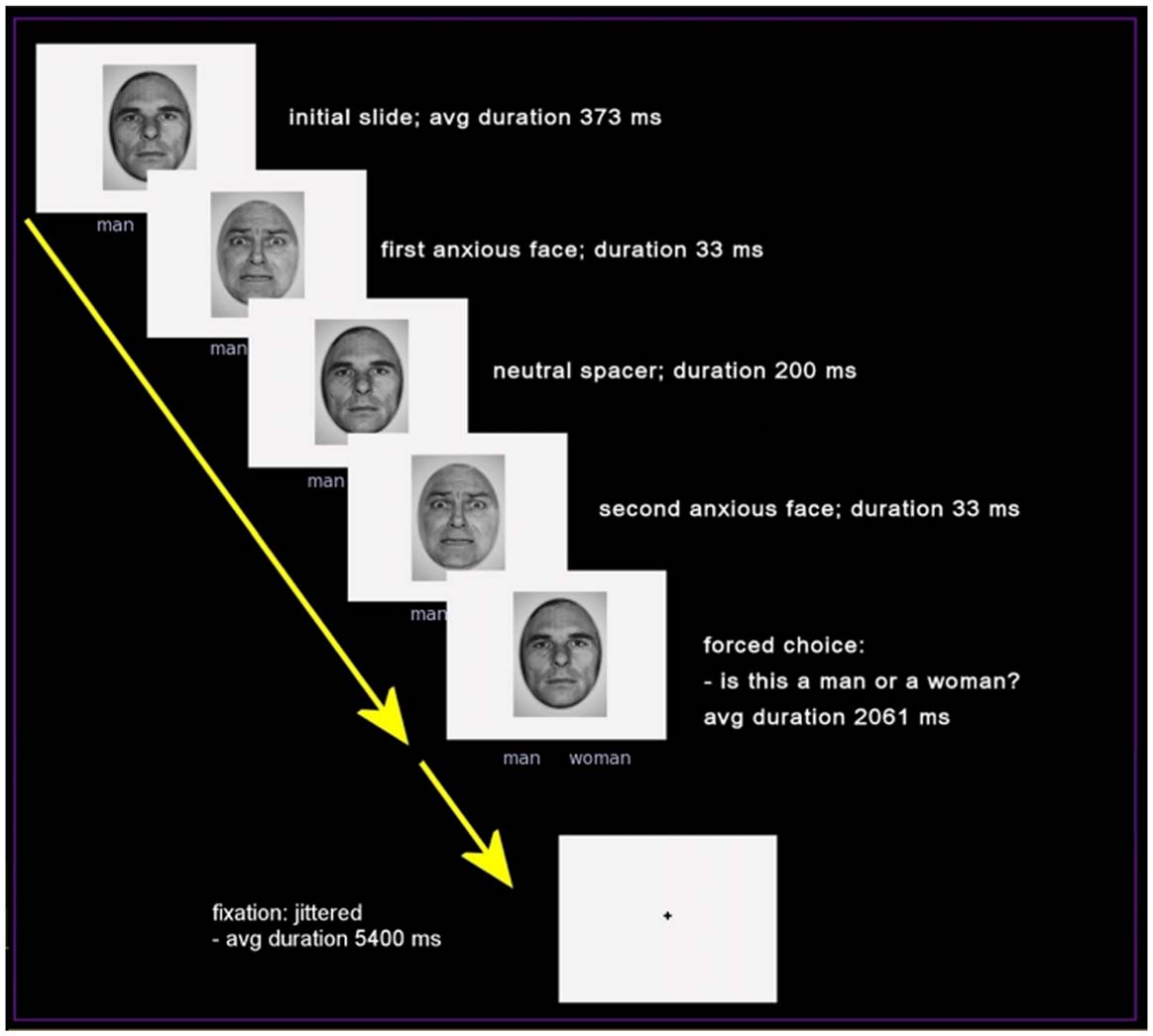
Design of backward masking trials used in the fMRI study. From trial onset the subject is presented with a male or female face. Inserted into this presentation are two subthreshold presentations of different corresponding male or female face. Each trial began with the presentation a neutral face. After an average of 373 msec, a 33 msec anxious face appeared followed by the reappearance of the neutral face for 200 msec. A second 33 mec presentation of the anxious face occurred followed by the final presentation of the neutral face. Participants were asked to identify if the neutral face was a man or a woman. Each trial was followed by a fixation screen for an average of 5400 msec.

In the backward masking task the subject was asked to look at a neutral face (the mask), and decide if it was a man or a women (see Figure 1). Below the face image, the words “man” and “woman” appeared to the left and right of center, respectively. The subjects pressed two buttons of a fiber optic response pad to select either “man” or “woman”. When selected, the text changed in color from black to blue to indicate the subject's choice. Subjects were asked to respond as quickly and accurately as they could. The total time for all face trials was 2700 msec. During each trial a single neutral face of a man or woman was presented. Interleaved into this presentation were two subthreshold (33 msec) presentations of an anxious target face. The onset of the first flash of an anxious face was varied between 173 and 573 msec. from the beginning of a trial. A fixed period of 200 msec. followed with the redisplay of the neutral face. This was followed by the second 33 msec. presentation of the anxious face and then the final redisplay of the neutral mask 1861 to 2061 msec. to finish out the trial. Each face trial was followed by a fixation screen. The duration of this inter-stimulus fixation interval was normally distributed with the average time equal to 5400 msec and a range of 2700 to 10800 msec. The order of the trials was randomized within participants and some stimuli were repeated. A total of 96 trials was presented and the total time of the experiment was 12 minutes. Reaction times and errors were recorded. Image presentation and response recording were done using E-Prime v1.2 (Psychology Software Tools, Pittsburgh, PA).

Images were acquired using a GE 3T whole body short bore scanner with 8 parallel receiver channels (General Electric, Milwaukee, WI). A three-dimensional volume SPGR pulse sequence with 124 slices (1.5 mm thick) was used to acquire anatomical images in the axial plane. Functional images were acquired with an optimized gradient-echo EPI sequence, and covering 13 axial slices (3 mm thick, no gap), beginning just below the most ventral part of the inferior temporal cortices (bilaterally) and encompassing the entire amygdala (TR/TE = 2700/35 ms, FOV = 24 cm, matrix  = 64×64, flip angle 90°). Acquired images were transferred to a workstation, preprocessed and analyzed using Brain Voyager QX version 1.8.6 (Brain Innovation B.V., Maastricht, The Netherlands). The functional data sets were slice-time corrected, linear detrended, 3D-motion corrected and realigned (all using sinc-interpolation), and normalized to Talairach space [42]. High-resolution T1-weighted three-dimensional (3D) anatomical MR data sets were transformed into Talairach space, used for co-registration and averaged to generate a composite image onto which functional activation results were projected. Given our a priori hypotheses, regions of particular interest were the amygdala and fusiform brain areas given their role in fear coding [43] and face processing [5], respectively. A standard Brodmann map (Brain Voyager QX) was co-registered to the average composite anatomic data set and used to prescribe Regions of Interest (ROI) in the right and left amygdala and fusiform gyri (Brodmann Areas 37 and 19).

An event related deconvolution model for each participant was used to examine BOLD signal at each and every voxel within the ROI. Using a random-effects analysis the backward masked anxious face trials and fixation cross conditions were set as the explanatory variables accounting for differences in blood oxygen level dependent signals within and between groups. Contrasts were corrected for multiple-comparisons using the false discovery rate methodology .05 [44], and the average statistical value for ROI are reported. Finally the time course for the group average % BOLD signal change relative to the onset of the anxious face stimuli was plotted.

These individual contrast images were then used in second-level random effects models that account for both scan-to-scan and subject-to-subject variability, and to determine task specific localized responses at the group level.

### Preliminary Test Series

A preliminary series was undertaken to replicate the backward masking findings for anxious face stimuli reported in the literature using our stimuli. In the preliminary test series all details regarding the stimulus preparation, display times, intertrial jitter remained the same. The only difference was that in one half of the trials a neutral face was presented as the target stimulus and the other half an anxious face was displayed. The neutral faces were selected from a battery of 16 male and 16 female faces that had been morphed with happy photographs of the same individuals such that the final face details were shifted 20% toward happy. This subtle shift in the neutral facial characteristics was applied because it has been suggested that neutral faces can appear somewhat negative, cold and threatening [45]. In the preliminary test series 3 healthy young women were scanned. Each participant underwent two full backward mask series in which the presentation of anxious target trials and neutral target trials was randomized. The data from these series was analyzed using an event related deconvolution analysis and involved ROI prescribed in the right and left amygdala and fusiform gyri. These data were then cluster threshold corrected for multiple comparisons.

## Results

### Preliminary Test Series

In our preliminary scan series we presented backward masked anxious and neutral faces in a typically developed independent group. We found greater activation in the amygdala and fusiform, bilaterally, to anxious compared to neutral faces [Right Amygdala (20, −3. −15) 254 voxels: t(1359)–2.62, p<0.026; Left Amygdala (−22 −7 −20) 810 voxels: t(1359) = 3.035, p<0.011. Right Fusiform: (41, −41, 30) 356 voxels; t(1359) = 3.836 p<0.004: Left Fusiform (−44, −45,−18) 946 voxels; t(1359) = 4.56 p<0.001]. These findings are consistent with previous work identifying heightened amygdala activation to rapid subthreshold presentations of anxious and neutral faces [33].

### ASD Pilot Study: Participant Debriefing

Upon the completion of their scan participants were asked to describe the presented stimuli. None of the participants reported seeing anxious flashes of faces. However, some did note the presence of a “flash” during the trials, but were not able to expand their description of what had been perceived.

### Behavioural Performance

**Figure 2:**
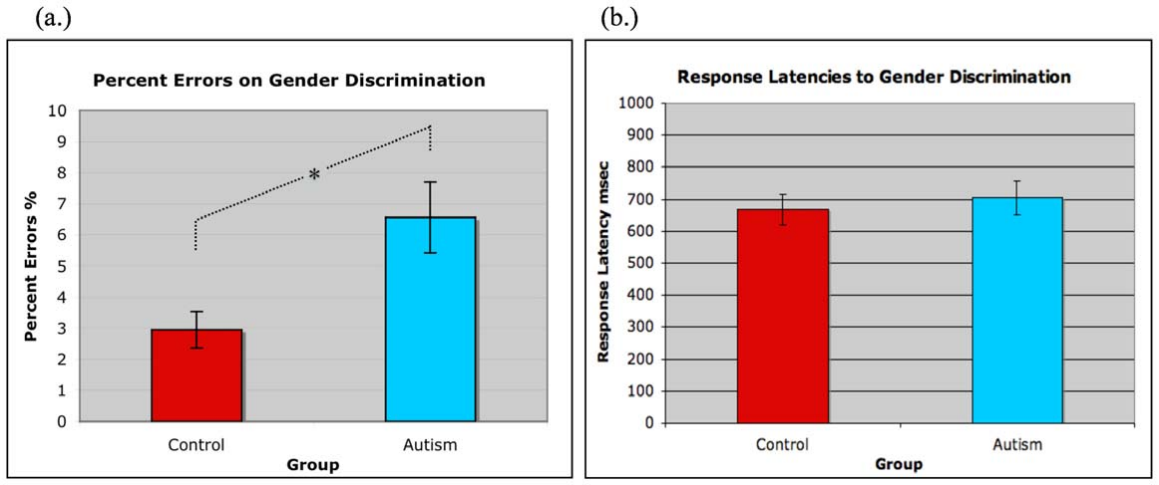
Performance data for gender discrimination of neutral mask faces. Performance by individuals with ASD (blue) and controls (red) was associated with mean group percent errors (a.) and response latencies (b.). The asterisk (*) indicates significant group differences.

The mean percent correct responses for gender discrimination was 93.44%+3.95 for the adults with ASD and 97.04%+2.01 for Controls. An independent samples t-test identified significantly higher error rates for the adults with ASD than controls t(22) = 2.81, p<.011 (Figure 2a).

The mean latency to response on gender discrimination trials was 704 msec (SD ±188) for adults with ASD and 669 ms (SD ±165) for Controls. No group differences in response latency were identified in an independent samples t-test (t(22) = 0.48 p<0.63) (Figure 2b).

### Functional analyses of ROIs

**Figure 3:**
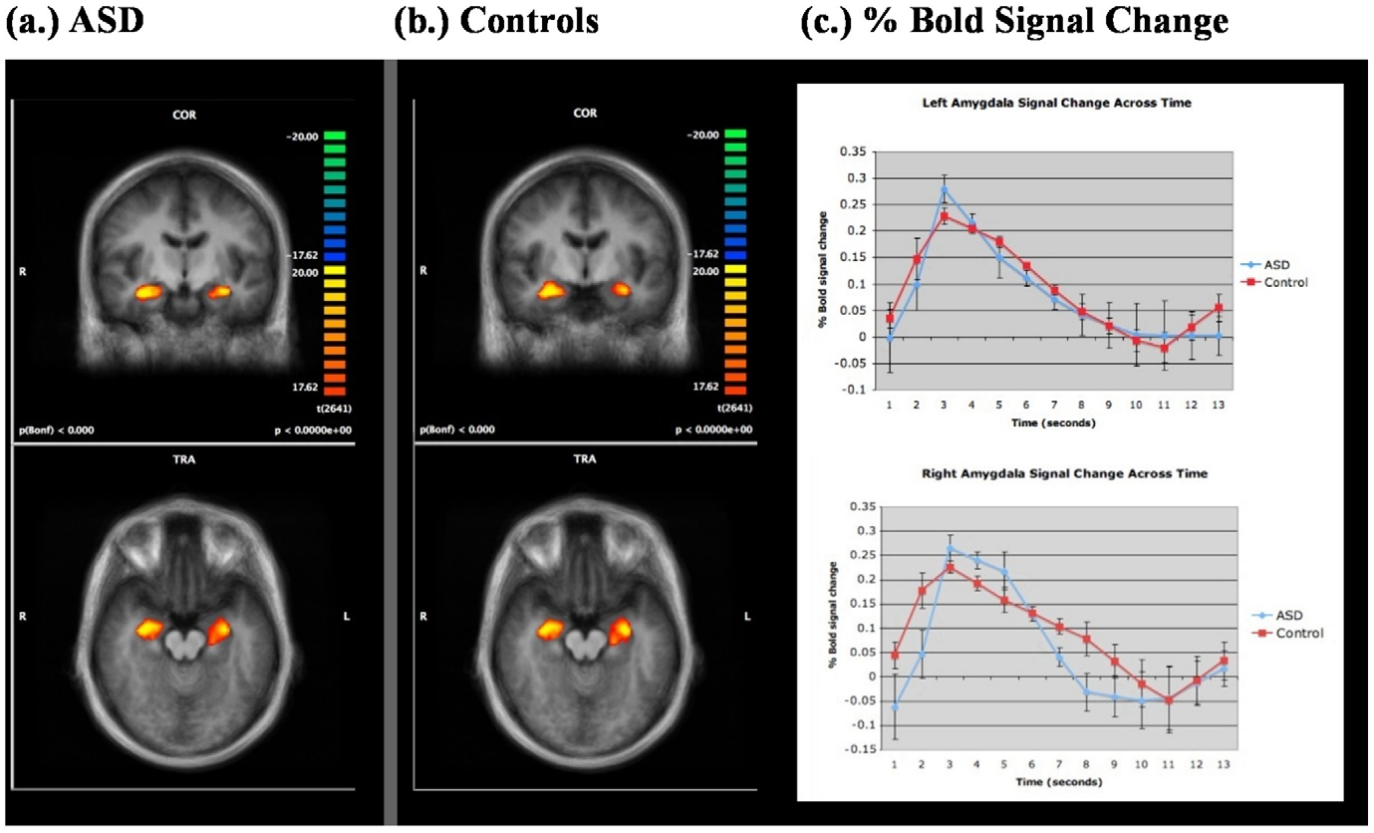
Amygdala activation during backward masking trials. Statistical maps of a priori regions of interest defined using the Talairach Atlas and superimposed on a composite average of 24 anatomical T1 image sets normalized to Talairach space. Note bilateral amygdala activation in both individuals with ASD (a.) and age-matched controls (b.) during the presentation of subthreshold anxious face stimuli. Images are presented according to radiological convention. Mean peristimulus plots of the average estimated hemodynamic responses to subthreshold anxious face images are shown (c.) for the right and left amygdala in individuals with ASD and controls. The control group is shown in red, and the ASD group is shown in blue.

**Figure 4:**
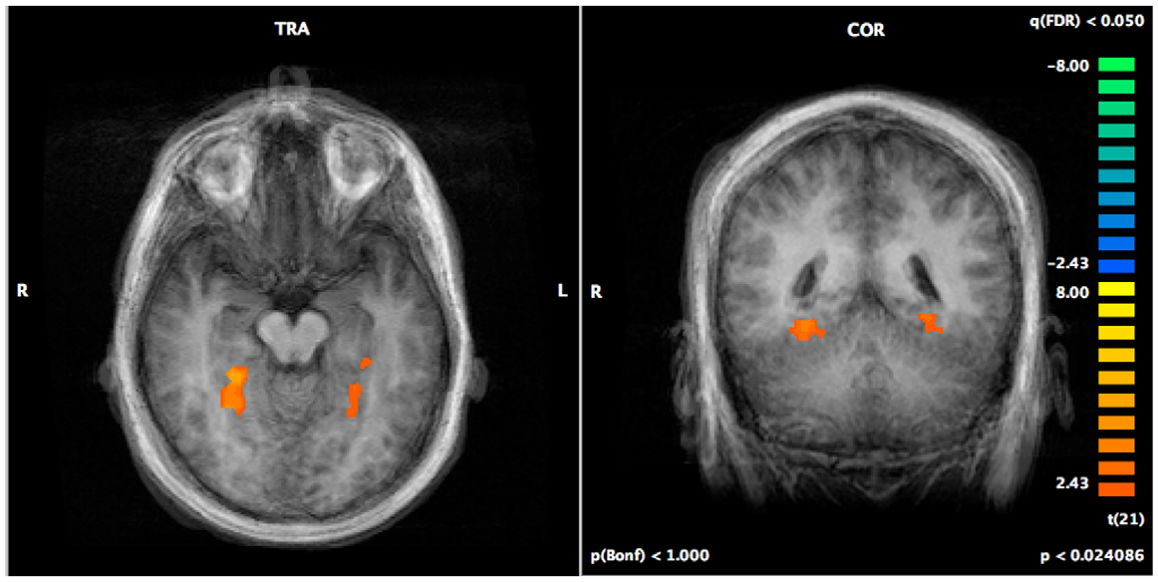
Fusiform activation during backward masking trials – between group comparison.

After correction for multiple comparisons, within group t-maps of the functional data for individuals with ASD and typically developing individuals identified significant bilateral amygdala activation during the subconscious presentation of anxious faces (all values <0.001) (Fig. 3.) in all subjects. Examining between group differences using a random effects analysis, no significant group differences were found in amygdala activation between the ASD and control groups (Table 2.). The average % BOLD signal change calculated for the right and left Amygdala ROIs (Figure 3c) identified that the engagement of the amygdala by both the ASD and control groups followed similar time courses. Finally, significant between group differences were identified in the fusiform region, bilaterally, with controls showing greater activation of the region than individuals with ASD (Table 3.; Figure 4.).

**Table 2:** Amygdala activation during backward masking trials – within group comparison of backward masked face trials versus fixation periods.

				Talairach Coordinates		
Within-group Comparison	Brain Region	t values (11)	p value	x	y	z
ASD	Right Amygdala	13.520	*0.001	25	−9	−18
	Left Amygdala	13.150	*0.001	−22	−5	−11
Controls	Right Amygdala	11.173	*0.001	23	−8	−16
	Left Amygdala	11.350	*0.001	−20	−4	−12

Data presented are the t- and p- values for amygdala activation within the individuals with ASD group (ASD) and within the typically developed controls group (controls). The asterisk (*) indicates significant values. Contrasts were corrected for multiple-comparisons using the false discovery rate of q = 0.05.

*FDR: q = 0.05.

**Table 3:** Random fixed effects analysis examining between group differences revealed greater bilateral recruitment of the fusiform gyrus in controls compared to individuals with ASD.

				Talairach Coordinates		
Between-group comparison	Brain Region	t values (22)	p value	x	y	z
Control < > ASD	Right Amygdala	1.050	0.305	23	−62	−7
	Left Amygdala	0.989	0.333	−20	−63	−7
Control > ASD	Right Fusiform BA37	5.635	*0.001	28	−46	−13
	Left Fusiform BA37	3.718	*0.001	−27	−47	−14

No group differences were observed between individuals with ASD and controls in amygdala activation. The asterisk indicates significant values. Contrasts were corrected for multiple-comparisons using the false discovery rate of q = 0.05.

## Discussion

In the present pilot work we found that the presentation of backward-masked anxious faces was associated with heightened neural activation of the amygdala in both high functioning adults with ASD and matched controls. Unexpectedly, neither the intensity nor the time-course of amygdala activation distinguished the groups. While these findings fail to replicate some previous reports of amygdala hypoactivation in ASD during the processing of faces and emotions, they appear consistent with reports of normal or hyper- activation of the amygdala when factors such as the length of gaze dwell time or gaze direction are included in imaging analyses [32], [46]. In the present study, subjects were actively engaged in the processing necessary for gender discrimination (neutral mask faces) when the sub-threshold flashes of anxious faces occurred. Behaviourally, no group differences were observed in the latencies to response for the gender discrimination, again suggesting that both groups were actively processing the face stimuli across the same time interval. This design, therefore, provided less opportunity for subjects to shift their gaze and permitted an examination of amygdala recruitment associated with the rapid sub-threshold presentation of the anxious faces.

Some have suggested that the amygdala is capable of alerting the cortex to emotionally salient information by virtue of the fast-conducting sub-cortical magnocellular pathway [10], [36]. Visual information that is low in spatial resolution [47]-[49] is conveyed along projections linking the superior colliculus to the thalamic pulvinar [50] and, in turn, linking the pulvinar to the amygdala [51]. Observations that these structures are engaged during the implicit processing of fearful face expressions [14], [33] has lead Morris and associates [52] to propose that this pathway is important in relaying fear relevant signals to the amygdala. Indeed, our results may suggest that in ASD, some aspects of the feedforward processing along this stream are preserved for emotionally salient information like threat or anxiety. Consistent with this notion are findings of amygdala dependent fear conditioning and comparable startle responses in individuals with ASD and controls [53]. Moreover, contrast-detection thresholds for flickering stimuli are normal in autism and thus signify patent magnocellular transmission [54], [55].

Group differences did emerge, when considering the response of the fusiform region to the face stimuli. Here, the adults with ASD showed significantly lower levels of fusiform activation during the backward masked trials compared to controls. This finding is consistent with a large body of evidence linking autism to fusiform hypoactivation across a wide variety of face processing tasks. [5], [27] Additionally, van Kooten et al., [56] recently identified histological evidence of abnormal neuron densities and neuron cell numbers in the fusiform region in seven patients with autism patients. Dense reciprocal connections link the amygdala with areas of the ventral visual processing stream, including the fusiform region [57] and may provide the mechanism through which the amygdala augments the processing of highly socially salient information, like faces.[5] Overall, our findings suggest that in ASD, while the amygdala can be actively engaged by highly socially salient information in the environment like anxious faces, the signaling of this information to structures downstream may be impoverished, and the pathways that facilitate subsequent processing deficient.

This study had number of limitations. First, contrasts were drawn against a baseline fixation condition, and only anxious faces were used as backward masked stimuli. As a consequence, it is not possible to distinguish brain activation associated with anxious face presentations from the performance of the gender discrimination task. In the design of the present study, this specificity was sacrificed in order to carry out the many repetitions needed to support group comparisons, and to collect event related data in the MRI scanner over a period of time that was tolerable and comfortable for our study group. This restriction was considered reasonable given our preliminary test findings and the large number of studies in the literature that have used gender discrimination as a baseline task [58]–[61] and the heightened amygdala activation reported with anxious face stimuli, with [62], [59] and without [63], [64] particular use of neutral face discrimination as a baseline condition. However, given the robust amygdala response observed in the present study, an expansion of this pilot could address this limitation by using both neutral and anxious faces as backward masked stimuli.

Another limitation of this work concerns the selection of high functioning adults with ASD. Dawson and associates have found electrophysiological evidence (event related potential) that the speed of neural responses to fear faces is associated with joint attention and social processing capacities in autism, and that children with low social capabilities show particularly slowed posterior n300 latencies to fear faces. The present findings cannot be extrapolated the broad Autism spectrum, and further work is needed to explore amygdala function across a range of intellectual abilities.

In sum, the present findings appear to conflict with a neurofunctional model of autism that places the locus of impairment in the amygdala. However, our results are consistent with views that emphasize changes in the modulation of activity [29] or hyper/hypo-connectivity [65] across the neurofunctional network that supports social/emotional processing. We found that amygdala activation associated with the presentation of rapid subthreshold anxious face stimuli in individuals with autism is indistinguishable from that seen in normal controls. Yet, under such stimulus conditions, activation within the fusiform region is reduced in individuals with ASD compared to typical controls. Importantly, further work is needed to substantiate these pilot findings in a larger study sample. Still, our findings suggest that in ASD, while the amygdala can be engaged by the transmission of highly salient social information along subcortical routes, the subsequent recruitment of the reciprocally connected regions (eg. the fusiform gyri) is deficient. This work may have important implications regarding symptoms of comorbid anxiety in ASD that are commonly reported clinically [53]. Activation of the amygdala without concurrent downstream processing may leave the individual with ASD in a generalized state of preparedness and lacking in the information necessary to resolve the complexities of their social environment. Moreover, the developmental impact of impoverished reciprocal feedback may impact on the consolidation of specialized regions like the fusiform gyrus [5]. Nonetheless, our findings emphasize that irregularities in amygdala function need to be considered within the broader context of the social brain network.
